# Domestic

**Published:** 1841-05-22

**Authors:** 


### DOMESTIC.

Interments *in the City and Liberties of Philadelphia, from the* 8*th to the* 15*th of May*, 1841.

| Diseases. | Adults. | Children. | Diseases. | Adults. | Children. |
|---|---|---|---|---|---|
| Apoplexy, | 1 | 0 | *Brought forward,* | 35 | 57 |
| Casualty, | 1 | 0 | Indigestion, | 0 | 1 |
| Croup, | 0 | 4 | Measles, | 0 | 11 |
| Congestion of lungs, | 0 | 2 | Mortification, | 1 | 0 |
| Congestion of brain, | 0 | 2 | Old age, | 3 | 0 |
| Consumption of the lungs, | 11 | 6 | Osteo sarcoma, | 1 | 0 |
| Convulsions, | 0 | 4 | Palsy, | 1 | 0 |
| Coxalgia, | 0 | 1 | Pneumothorax, | 2 | 0 |
| Dropsy, | 1 | 0 | Rupture of heart, | 1 | 0 |
| —— head, | 0 | 8 | Small pox, | 0 | 1 |
| Disease of brain, | 0 | 1 | Still-born, | 0 | 9 |
| —— heart, | 1 | 0 | Suicide, | 1 | 0 |
| —— breast, | 0 | 1 | Syphilis, | 0 | 1 |
| Dysentery, | 1 | 0 | Trisnus, | 0 | 1 |
| Debility, | 0 | 4 | Ulceration of throat, | 1 | 0 |
| Erysipelas, | 1 | 0 | Unknown, | 2 | 0 |
| Enlargement of the heart, | 1 | 0 | Worms, | 0 | 1 |
| Fever, bilious, | 1 | 0 | Total, 130— | 48 | 82 |
| —— typhus, | 3 | 0 | | | |
| —— scarlet, | 0 | 4 | | | |
| Gout, | 1 | 0 | | | |
| Inflammation of the brain, | 1 | 6 | | | |
| —— bronchi, | 2 | 4 | | | |
| —— lungs, | 2 | 7 | | | |
| —— stomach, | 0 | 1 | | | |
| —— stomach and bowels, | 1 | 1 | | | |
| —— bowels, | 1 | 1 | | | |
| —— liver, | 1 | 0 | | | |
| —— uterus, | 1 | 0 | | | |
| —— peritonæum, | 2 | 0 | | | |
| Intemperance, | 1 | 0 | | | |
| *Carried forward,* | 35 | 57 | | | |

Of the above, there

| | |
|---|---|
| were under 1 year | 30 |
| From 1 to 2 | 18 |
| 2 to 5 | 24 |
| 5 to 10 | 6 |
| 10 to 15 | 2 |
| 15 to 20 | 2 |
| 20 to 30 | 13 |
| 30 to 40 | 12 |
| 40 to 50 | 7 |
| 50 to 60 | 1 |
| 60 to 70 | 6 |
| 70 to 80 | 5 |
| 80 to 90 | 2 |
| 90 to 100 | 1 |
| 100 to 110 | 1 |
| Total, | 130 |

Of the above there were 11 from the almshouse, 11 people of colour, and two from the country, which are included in the total amount.

*Died*, in this city, May 18, 1841, Dr. WILLIAM P. DEWEES, in the 75th year of his age.

